# MicroRNA‐301b‐3p contributes to tumour growth of human hepatocellular carcinoma by repressing vestigial like family member 4

**DOI:** 10.1111/jcmm.14361

**Published:** 2019-06-17

**Authors:** Yang Guo, Bowen Yao, Qiaojuan Zhu, Zunqiang Xiao, Linjun Hu, Xin Liu, Lijie Li, Jiahui Wang, Qiuran Xu, Liu Yang, Dongsheng Huang

**Affiliations:** ^1^ Graduate Department BengBu Medical College BengBu Anhui Province China; ^2^ Key Laboratory of Tumour Molecular Diagnosis and Individualized Medicine of Zhejiang Province Zhejiang Provincial People's Hospital (People's Hospital of Hangzhou Medical College) Hangzhou Zhejiang Province China; ^3^ Department of Hepatobiliary Surgery The First Affiliated Hospital of Xi'an Jiaotong University Xi'an Shaanxi Province China; ^4^ Department of Second Clinical Medical College Zhejiang Chinese Medical University Hangzhou Zhejiang Province China; ^5^ The Medical College of Qindao University Qindao Shandong Province China; ^6^ School of Basic Medical Sciences Shandong University Jinan Shandong Province China

**Keywords:** apoptosis, cell proliferation, G2/M phase arrest, hepatocellular carcinoma, miR‐301b‐3p, VGLL4

## Abstract

MicroRNAs (miRNAs) are key regulators in the tumour growth and metastasis of human hepatocellular carcinoma (HCC). Increasing evidence suggests that miR‐301b‐3p functions as a driver in various types of human cancer. However, the expression pattern of miR‐301b‐3p and its functional role as well as underlying molecular mechanism in HCC remain poorly known. Our study found that miR‐301b‐3p expression was significantly up‐regulated in HCC tissues compared to adjacent non‐tumour tissues. Clinical association analysis revealed that the high level of miR‐301b‐3p closely correlated with large tumour size and advanced tumour‐node‐metastasis stages. Importantly, the high miR‐301b‐3p level predicted a prominent poorer overall survival of HCC patients. Knockdown of miR‐301b‐3p suppressed cell proliferation, led to cell cycle arrest at G2/M phase and induced apoptosis of Huh7 and Hep3B cells. Furthermore, miR‐301b‐3p knockdown suppressed tumour growth of HCC in mice. Mechanistically, miR‐301b‐3p directly bond to 3′UTR of vestigial like family member 4 (VGLL4) and negatively regulated its expression. The expression of VGLL4 mRNA was down‐regulated and inversely correlated with miR‐301b‐3p level in HCC tissues. Notably, VGLL4 knockdown markedly repressed cell proliferation, resulted in G2/M phase arrest and promoted apoptosis of HCC cells. Accordingly, VGLL4 silencing rescued miR‐301b‐3p knockdown attenuated HCC cell proliferation, cell cycle progression and apoptosis resistance. Collectively, our results suggest that miR‐301b‐3p is highly expressed in HCC. miR‐301b‐3p facilitates cell proliferation, promotes cell cycle progression and inhibits apoptosis of HCC cells by repressing VGLL4.

## 1 INTRODUCTION

Hepatocellular carcinoma (HCC), the most common phenotype of liver cancer, is a growing public health concern worldwide, especially in China.^1^ According to cancer statistics published in 2017, liver cancer was the second most frequent cause of cancer death in males and the sixth in females around the world.^2^ Unfortunately, due to the lack of effective early diagnosis, most HCC patients are diagnosed at an advanced stage, which is one of the main causes for poor prognosis.^3^ On the other hand, the complex mechanism involved in hepatocarcinogenesis limits the development of targeted drug for advanced HCC. Therefore, there is an urgent need to explore novel diagnostic biomarkers and find more effective treatments for HCC.

MicroRNAs (miRNAs) are a subset of conserved, single‐strand, small non‐coding RNAs (18‐24nt), which can regulate gene expression by base‐pairing to the 3′‐UTR of the target mRNA and subsequently changing mRNA degradation and/or translational dysfunction.^4^, ^5^ Accumulating studies have found that miRNAs are aberrantly expressed and dysfunctional in HCC.^6^, ^7^, ^8^, ^9^ Recently, miRNAs have been shown to be associated with diagnosis, development, progression and prognosis of human HCC.^10^, ^11^, ^12^, ^13^ miR‐301b‐3p is recently recognized as a cancer‐related miRNA. The aberrant expression and dysregulation of miR‐301b‐3p has been detected in various types of cancer, including oesophageal adenocarcinoma,^14^ pancreatic cancer,^15^, ^16^ breast cancer,^17^, ^18^ acute myeloid leukaemia,^19^ bladder cancer,^20^ lung cancer,^21^ prostate cancer^22^ and melanoma.^23^ In triple‐negative breast cancer, miR‐301b‐3p is highly expressed and enhances cell proliferation and apoptosis resistance by repressing cylindromatosis (CYLD).^18^ Furthermore, increased expression of miR‐301b‐3p indicates poor survival of patients with pancreatic cancer, and contributes to epithelial‐to‐mesenchymal transition (EMT) and cancer cell resistance to the gemcitabine by targeting nuclear receptor subfamily 3 group C member 2 (NR3C2).^16^ Overexpression of miR‐301b‐3p is a frequently even in lung cancer and facilitates cell growth, apoptosis resistance and resistance of cells to chemotherapy via suppressing Bim.^21^ Moreover, miR‐301b‐3p functions as an oncogene in pancreatic cancer via contributing to cell migration, invasion and gemcitabine resistance by targeting TP63.^15^ But, the expression pattern, functional role and potential mechanism of miR‐301b‐3p in HCC remain poorly known.

In this study, we aimed to explore the clinical significance of miR‐301b‐3p and its function as well as molecular mechanism in HCC. Our findings indicated that miR‐301b‐3p might be considered as a potential prognostic marker and contributed to tumour growth of human HCC by targeting vestigial like family member 4 (VGLL4).

## 2 MATERIALS AND METHODS

### 2.1 Patients and tissue samples

The HCC and matched tumour‐adjacent normal liver tissues were obtained in The First Affiliated Hospital of Xi'an Jiatong University during surgery. All specimens were pathologically confirmed as HCC. No patient received pre‐operative treatments, such as radiofrequency ablation, transarterial chemoembolization, immunotherapy and targeted therapy. These specimens were snap frozen in liquid nitrogen and then stored at −80°C. The clinical information of HCC patients was shown in Table 1. All patients signed the informed contents. This study was examined and approved by the Ethic Committee of The First Affiliated Hospital of Xi'an Jiatong University.

**Table 1:** Correlation between miR‐301b expression and clinicopathologic characteristics in hepatocellular carcinoma

Characteristics	n = 80	miR‐301b		*P*
		Low expression (n = 40)	High expression (n = 40)	
Age (y)				
<50	35	15	20	0.260
≥50	45	25	20	
Sex				
Male	63	30	33	0.412
Female	17	10	7	
HBV				
Absent	28	17	11	0.160
Present	52	23	29	
Serum AFP level (ng/mL)				
<20	27	17	10	0.098
≥20	53	23	30	
Tumour size (cm)				
<5	26	18	8	0.017^a^
≥5	54	22	32	
No. of tumour nodules				
1	65	34	31	0.390
≥2	15	6	9	
Cirrhosis				
Absent	35	21	14	0.115
Present	45	19	26	
Venous infiltration				
Absent	44	25	19	0.178
Present	36	15	21	
Edmondson‐Steiner grading				
I+II	57	31	26	0.217
III+IV	23	9	14	
TNM tumour stage				
I+II	63	36	27	0.014^a^
III+IV	17	4	13	

AFP: alpha‐fetoprotein; HBV: hepatitis B virus; TNM: tumour‐node‐metastasis.

a Statistically significant.

### 2.2 Cell culture and transfection

Human HCC cell lines Huh7 and Hep3B were maintained in our lab and cultured as previously described.^6^ The precursor miR‐301b‐3p in non‐viral vectors, lentivector‐mediated miR‐301b‐3p inhibitors and corresponding control clones were obtained from GeneCopoeia Inc. (Guangzhou, China). pcDNA3.1‐VGLL4 was constructed as previously described.^24^ VGLL4 siRNA (5′‐CAACGACCACGUCUCCAAAtt‐3′) and scrambled siRNA (5′‐ACAGACUUCGGAGUACCUG‐3′) were designed and purchased from RiboBio (Guangzhou, China). Lipofectamine^®^ 2000 Reagent (Invitrogen, Carlsbad, CA, USA) was used for vector transfection according to the manufacturer's protocols.

### 2.3 RNA extraction and quantitative real‐time PCR

Total RNA was extracted from tissue samples and cells using TRIzol reagent (Invitrogen). The RNA was reverse‐transcribed into cDNA using TaqMan miRNA reverse transcription (Applied Biosystems, Foster City, CA, USA) and a PrimeScript Reverse Transcriptase kit (Takara, Shiga, Japan). The expression levels of miR‐301b‐3p and VGLL4 mRNA were measured using miRNA‐specific TaqMan miRNA Assay Kit (Applied Biosystems) and the SYBR Premix Ex Taq™ Kit (Takara) in the Applied Biosystems 7500 Sequence Detection system as previously described.^25^ The primer sequences of miR‐301b‐3p (RT: 5′‐GTC GTA TCC AGT GCA GGG TCC GAG GTA TTC GCA CTG GAT ACG ACG TCA CGT‐3′, forward: 5′‐TCC GAC GAA ACT GTT ATA GTA‐3′ and reverse: 5′‐GTG CAG GGT CCG AGG T‐3′), VGLL4 (forward: 5′‐TTG TCC TAG GAA ACG GGC TG‐3′ and reverse: 5′‐GGG CTT ACT GGT AGA CGG TG‐3′), GAPDH (forward: 5′‐CCA TGT TCG TCA TGG GTG TG‐3′ and reverse: 5′‐GGT GCT AAG CAG TTG GTG GTG‐3′) and U6 (forward: 5′‐GCT TCG GCA GCA CAT ATA CTA AAA T‐3′ and reverse: 5′‐CGC TTC ACG AAT TTG CGT GTC AT‐3′) were used.

### 2.4 Cell proliferation analysis

Cell proliferation was detected using Cell Counting Kit‐8 (CCK‐8) assay. CCK‐8 solution (10 μL, Dojindo Molecular Technologies, Inc., Kumamoto, Japan) was used for this assay as previously described.^6^

### 2.5 Colony formation assay

Cells that were transfected with corresponding vectors for 48 hours were harvested and seeded into 6‐well plates (500 cells/well). Then cells were incubated in complete culture medium for 12 days. The colonies were fixed with 4% paraformaldehyde for 30 minutes, and stained with 0.5% crystal violet for 30 minutes at room temperature.

### 2.6 Flow cytometry analysis

Cell cycle distribution and apoptosis were measured using a FACSCanto II flow cytometer (BD Biosciences, San Jose, CA, USA). PI/RNase Staining Buffer (BD biosciences) and the PE Annexin V Apoptosis Detection Kit I (BD biosciences) were used for these assays as previously described.^6^

### 2.7 Western blot analysis

The transfected HCC cells were lysed by protein lysis buffer (Beyotime, Shanghai, China) and the concentrations of protein was measured using a Pierce BCA protein assay kit (Tiangen Biotech Co., Ltd., Beijing, China). A total of 20‐30 μg protein was separated by 10% SDS‐PAGE and electro‐transferred to polyvinylidene fluoride membranes (Millipore, Billerica, MA, USA). After being blocked with 5% skimmed milk, the membranes were incubated with VGLL4 (ab140290; Abcam, Cambridge, MA, USA) and GAPDH antibodies (ab181602; Abcam) overnight at 4°C. After incubation with the horseradish peroxidase‐conjugated IgG secondary antibodies (Santa Cruz Biotechnology, Santa Cruz, CA, USA). The protein expression levels were detected by ECL reagent (Millipore) and imaged using Amersham Imager 600 from GE Healthcare Life Sciences (Beijing, China). The blots were semi‐quantified by ImageJ software (1.46; National Institutes of Health, Bethesda, MD, USA).

### 2.8 Luciferase reporter assays

The pEZX‐MT06 reporter containing wild type (wt) 3′UTR of VGLL4 was purchased from GeneCopoeia Inc. The mutation (mt) site of seed sequence was designed on wt 3′UTR of VGLL4 using a QuikChange II Site‐Directed Mutagenesis Kit (Agilent Technologies, La Jolla, CA, USA). Huh7 cells were co‐transfected with wt or mt reporter vectors and precursor miR‐301b‐3p or miR‐301b‐3p inhibitors. After 48 hours transfection, the dual‐luciferase reporter assay system (Promega, Madison, WI, USA) was used to examine the luciferase activity. Firefly luciferase activity was normalized to renilla luciferase activity.

### 2.9 *In vivo* tumour xenograft experiments

A total of 20 male BALB/c nude mice (4‐6 weeks old, 20‐25 g) were purchased for in vivo tumour xenograft experiments. Mice were divided into four group (n = 5 per group). miR‐301b‐3p inhibitors transfected Huh7 or Hep3B cells (1 × 10^6^ cells per injection) and control cells were subcutaneously injected into the left flank of mice. Tumours were measured once every 3 days, and the tumour volume was calculated using the formula: tumour volume (mm^3^) = (L × W^2^)/2, where L = long axis and W = short axis. The mice were killed by cervical dislocation under anaesthesia and the xenograft tumour tissues were harvested 3 weeks after implantation and subjected to immunohistochemistry for Ki‐67 staining.

### 2.10 Statistical analysis

Data were expressed as mean ± SD and analysed by GraphPad Prism 6.0 (GraphPad Inc., San Diego, CA, USA). Chi‐squared test was performed to determine the clinicopathological correlation of miR‐301b‐3p expression. Student's *t* test was performed between two groups or one‐way ANOVA followed by Tukey post hoc test was analysed for multiple comparison. Kaplan‐Meier method and log‐rank test were used to examine overall survival. Pearson correlation test was carried out to confirm the correlation between miR‐301b‐3p and VGLL4 mRNA expression in HCC tissues. A *P* < 0.05 was considered to be statistically significant.

## 3 RESULTS

### 3.1 High expression of miR‐301b‐3p correlates with poor prognosis of HCC patients

**Figure 1:**
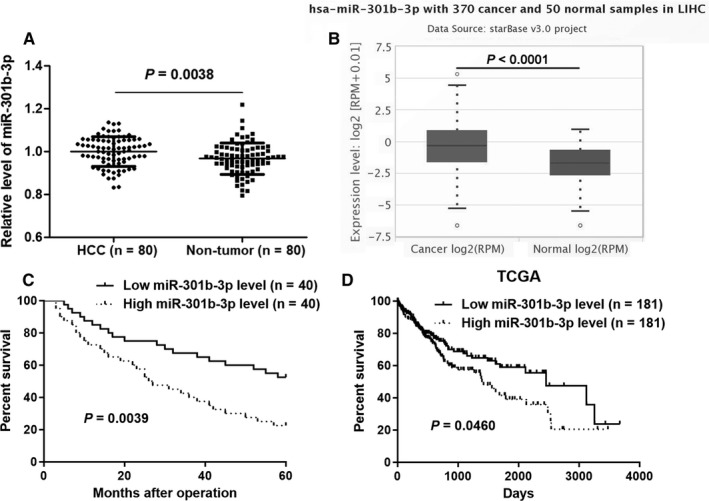
The expression and prognostic value of miR‐301b‐3p in hepatocellular carcinoma (HCC). A, The expression of miR‐301b‐3p was detected by qRT‐PCR between HCC tissues (n = 80) and adjacent non‐tumour tissues (n = 80). *P* = 0.0038 by *t* test. B, Analysis of HCC (n = 370) and normal liver samples (n = 50) in TCGA dataset from starBase V3.0 platform showed that miR‐301b‐3p was over‐expressed in HCC tissues. *P* < 0.0001 by *t* test. C, HCC patients were divided into two subgroups (low or high miR‐301b‐3p level) using the median level of miR‐301b‐3p as a cut‐off value. HCC patients with high miR‐301b‐3p level (n = 40) showed a significant poorer overall survival compared to low miR‐301b‐3p group (n = 40). *P* = 0.0039 by log‐rank test. D, TCGA data from starBase V3.0 platform indicated that low miR‐301b‐3p level predicted poor prognosis of HCC patients. Low miR‐301b‐3p level (n = 181), high miR‐301b‐3p level (n = 181), *P* = 0.0460 by log‐rank test

The expression of miR‐301b‐3p between HCC and corresponding tumour‐adjacent tissues was determined by quantitative real‐time PCR (qRT‐PCR). miR‐301b‐3p expression was significantly increased in HCC compared to matched paracancerous tissues (*P* = 0.0038, Figure 1A). Analysis of HCC and normal liver samples in TCGA Data Portal from starBase V3.0^26^ further confirmed that miR‐301b‐3p was highly expressed in HCC (*P* < 0.0001, Figure 1B). HCC cohort was divided into two subgroups (high or low miR‐301b‐3p group) using the median level of miR‐301b‐3p as cut‐off value. Clinical association analysis revealed that HCC patients with large tumour size and advanced tumour‐node‐metastasis (TNM) stages showed a higher expression of miR‐301b‐3p compared to patients with small tumour size and early TNM stages (*P* = 0.017 and 0.014, respectively, Table 1). Importantly, the follow‐up data indicated that HCC patients with high miR‐301b‐3p level showed a significant poorer overall survival compared to patients in low miR‐301b‐3p group (*P* = 0.0039, Figure 1C). Furthermore, survival analysis of miR‐301b‐3p in HCC from starBase V3.0^26^ demonstrated that elevated level of miR‐301b‐3p predicted poor prognosis of HCC patients (*P* = 0.0460, Figure 1D). Taken together, our data suggested that miR‐301b‐3p might be a promising prognostic biomarker for HCC patients.

### 3.2 miR‐301b‐3p knockdown inhibits HCC cell growth

**Figure 2:**
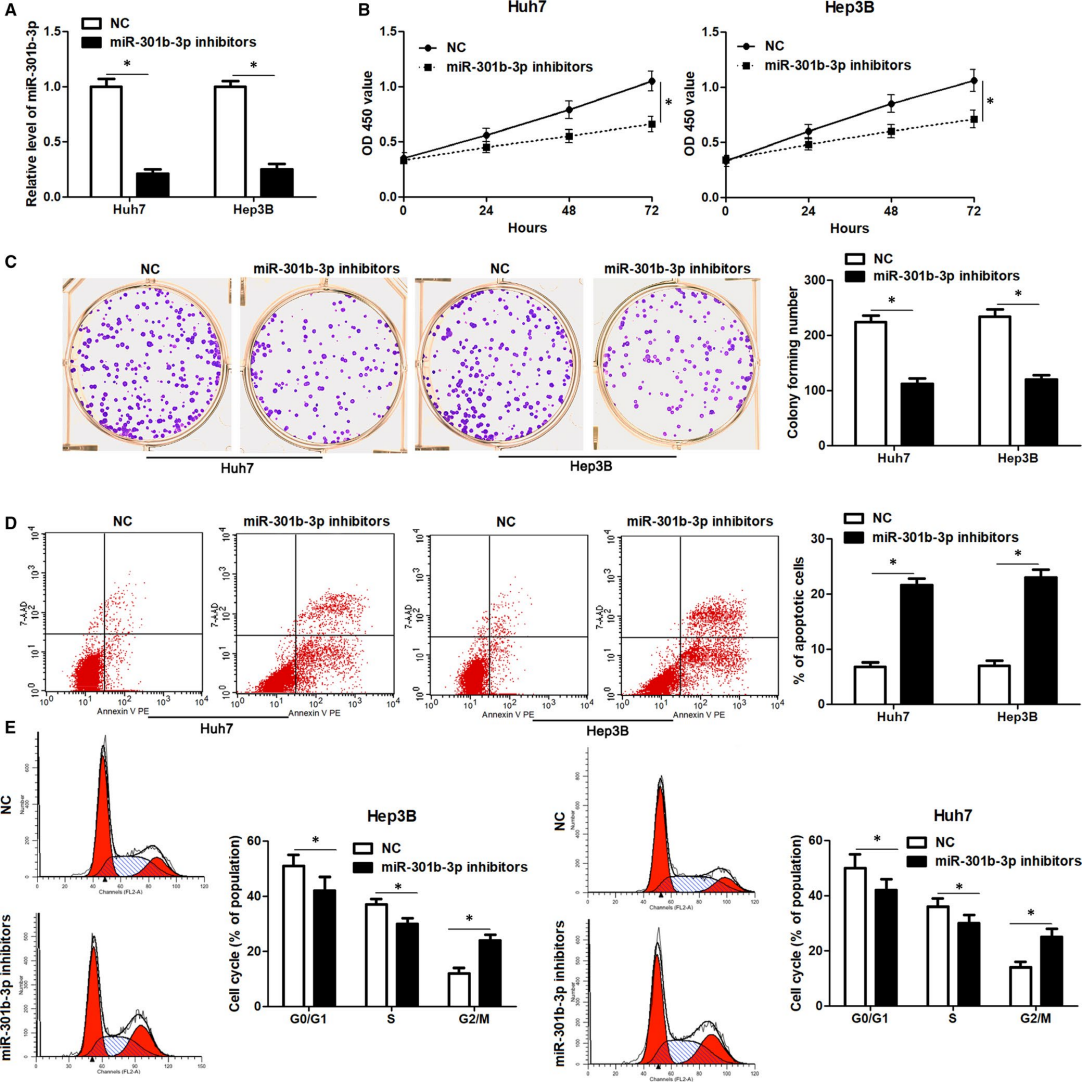
miR‐301b‐3p knockdown suppresses hepatocellular carcinoma (HCC) cell proliferation, results in G2/M phase arrest and induces apoptosis. A, miR‐301b‐3p inhibitors or negative control (NC) were transduced into Huh7 and Hep3B cells, and qRT‐PCR was performed to detect miR‐301b‐3p expression. n=three independent repeats, *P < 0.05 by *t* test. B and C, CCK‐8 and colony formation assays revealed that miR‐301b‐3p knockdown suppressed the proliferation of HCC cells. n = three independent repeats, *P < 0.05 by ANOVA or *t* test. D, The percentage of apoptotic HCC cells was significant increased after miR‐301b‐3p knockdown. n = three independent repeats, *P < 0.05 by *t* test. E, miR‐301b‐3p knockdown led to G2/M phase arrest in both Huh7 and Hep3B cells. n = three independent repeats, *P < 0.05 by *t* test

The expression pattern and clinical significance of miR‐301b‐3p indicated that miR‐301b‐3p might play an oncogenic role in HCC. Next, miR‐301b‐3p was knocked down by its inhibitors in both Huh7 and Hep3B cells (*P* < 0.05, Figure 2A). CCk‐8 and colony formation assay revealed that miR‐301b‐3p knockdown significantly reduced the proliferation of HCC cells (*P* < 0.05, Figure 2B and C). Moreover, flow cytometry assay indicated that miR‐301b‐3p knockdown markedly increased the percentage of apoptotic Huh7 and Hep3B cells (*P* < 0.05, Figure 2D). Cell cycle distribution analysis demonstrated that miR‐301b‐3p knockdown led to cell cycle arrest at G2/M phase in both Huh7 and Hep3B cells (*P* < 0.05, Figure 2E). Thus, miR‐301b‐3p acted an oncogenic role in HCC by regulating cell proliferation, cell cycle progression and apoptosis.

### 3.3 miR‐301b‐3p knockdown represses tumour growth of HCC in vivo

**Figure 3:**
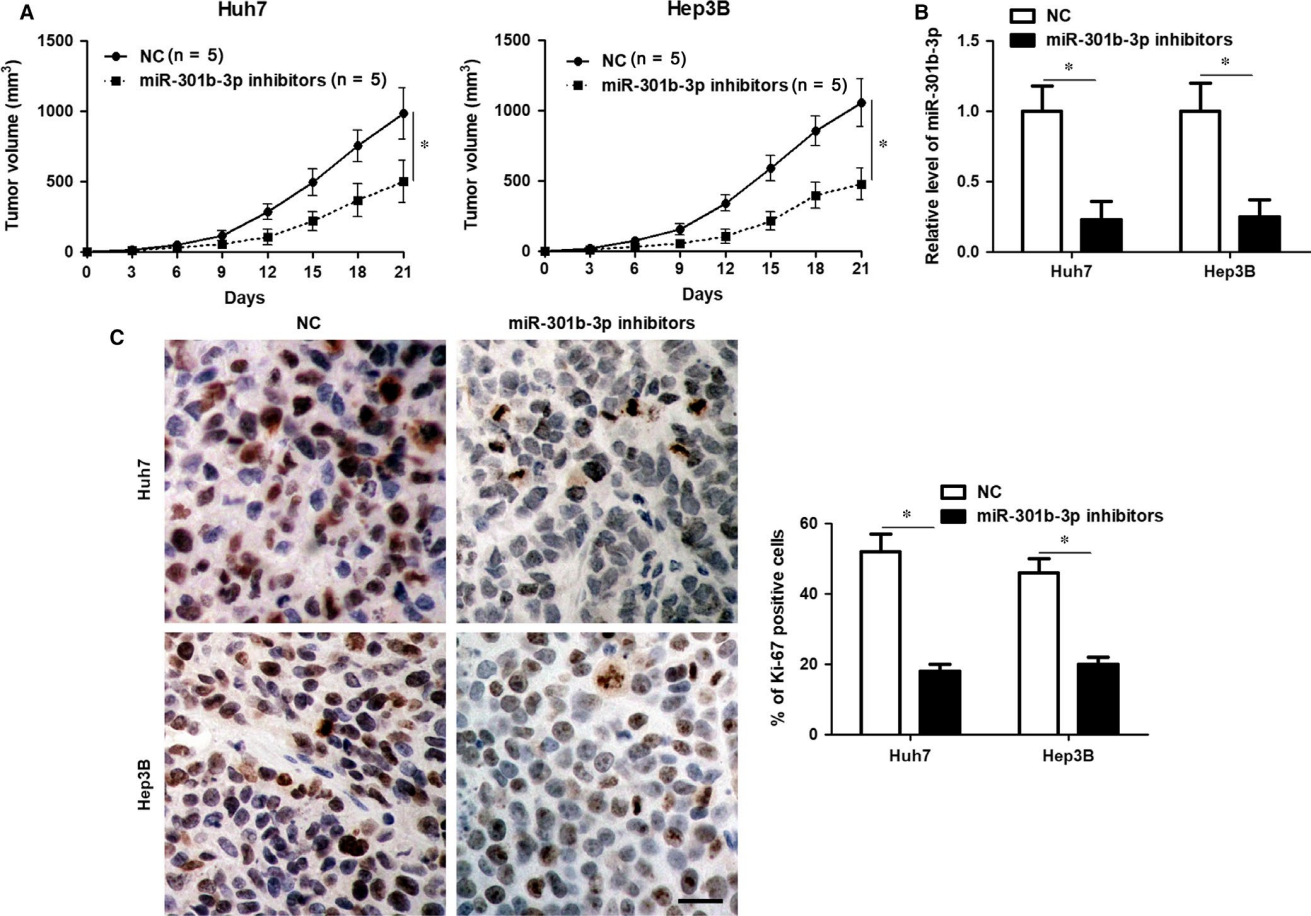
Knockdown of miR‐301b‐3p represses tumour growth of hepatocellular carcinoma (HCC) in vivo. A, Huh7 and Hep3B cells that were transfected with lentivector‐mediated miR‐301b‐3p inhibitors or negative control (NC) were implanted into nude mice. The tumour volume in miR‐301b‐3p knockdown group (n = 5) was markedly less compared to control group (n = 5). *P < 0.05 by ANOVA. B, The expression of miR‐301b‐3p in xenograft tumour tissues collected from miR‐301b‐3p knockdown group (n = 5) was prominently lower compared to control group (n = 5). *P < 0.05 by *t* test. C, Immunostaining of Ki‐67 indicated that the percentage of Ki‐67 positive tumour cells in xenograft tumour tissues collected from miR‐301b‐3p knockdown group (n = 5) was significantly lower compared to control group (n = 5). *P < 0.05 by *t* test. Scale bar: 50 μm

Huh7 and Hep3B cells with or without miR‐301b‐3p knockdown were used to construct the subcutaneous tumour formation model in mice. Tumour growth curves revealed that miR‐301b‐3p knockdown markedly restrained the growth of HCC cells in mice (*P* < 0.05, Figure 3A). The levels of miR‐301b‐3p in xenograft tumour tissues collected from miR‐301b‐3p knockdown group was significantly lower than those in control group (*P* < 0.05, Figure 3B). Furthermore, the percentage of Ki‐67 positive tumour cells in xenograft tumour tissues collected from miR‐301b‐3p knockdown group was also significantly lower than those in control group (*P* < 0.05, Figure 3C). Altogether, miR‐301b‐3p exerted tumour promoting role in HCC.

### 3.4 miR‐301b‐3p directly targets and regulates VGLL4 expression in HCC cells

**Figure 4:**
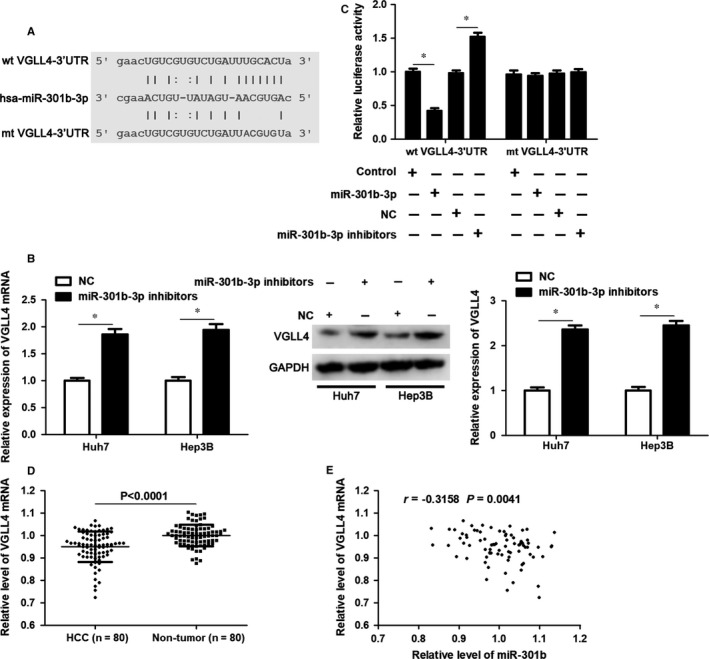
Vestigial like family member 4 (VGLL4) is identified as a novel target of miR‐301b‐3p. A, Bioinformatics analysis based on starBase V3.0 platform predicted VGLL4 as a candidate target of miR‐301‐3p. B, miR‐301b‐3p inhibitors or negative control (NC) were transduced into Huh7 and Hep3B cells, and qRT‐PCR and immunoblotting were performed to detect VGLL4 expression. n=three independent repeats, *P < 0.05 by *t* test. C, Wild type (wt) or mutated (mt) 3′UTR of VGLL4 and miR‐301b‐3p mimics or inhibitors were co‐transfected into Huh7 cells and the relative fluorescence intensity was detected. n=three independent repeats, *P < 0.05 by *t* test. D, The expression of VGLL4 mRNA was detected by qRT‐PCR between HCC tissues (n = 80) and adjacent non‐tumour tissues (n = 80). *P* < 0.0001 by *t* test. E, An inverse correlation between miR‐301b‐3p level and the expression of VGLL4 mRNA was found in HCC tissues. n = 80, *P* = 0.0041 by Pearson correlation test

To explore the molecular mechanism involved in the oncogenic role of miR‐301b‐3p, VGLL4 was predicted as a candidate target of miR‐301b‐3p using starBase V3.0 online platform (Figure 4A).^26^, ^27^ Accordingly, our data indicated that miR‐301b‐3p knockdown significantly increased the levels VGLL4 mRNA and protein in both Huh7 and Hep3B cells (*P* < 0.05, Figure 4B). Notably, overexpression of miR‐301b‐3p markedly decreased whereas miR‐301b‐3p knockdown significantly reduced the fluorescence intensity of vectors containing wt 3′UTR of VGLL4 (*P* < 0.05, Figure 4C). However, modulating miR‐301b‐3p level did not affect the luciferase activity of vectors containing mt 3′UTR of VGLL4 (Figure 4C). qRT‐PCR analysis revealed that the expression of VGLL4 mRNA was down‐regulated in HCC compared to matched tumour‐adjacent tissues (*P* < 0.0001, Figure 4D). The expression of VGLL4 protein in xenograft tumour tissues from miR‐301b‐3p knockdown group was significantly higher than that in control group (*P* < 0.05, Figure S1). Moreover, Pearson correlation analysis demonstrated that miR‐301b‐3p expression inversely correlated with the level of VGLL4 mRNA in HCC tissues (*r* = −0.3158, *P* = 0.0041, Figure 4E). This evidence verified that VGLL4 was a direct target of miR‐301b‐3p in HCC.

### 3.5 VGLL4 participates in miR‐301b‐3p knockdown‐induced growth arrest and apoptosis of HCC cells

**Figure 5:**
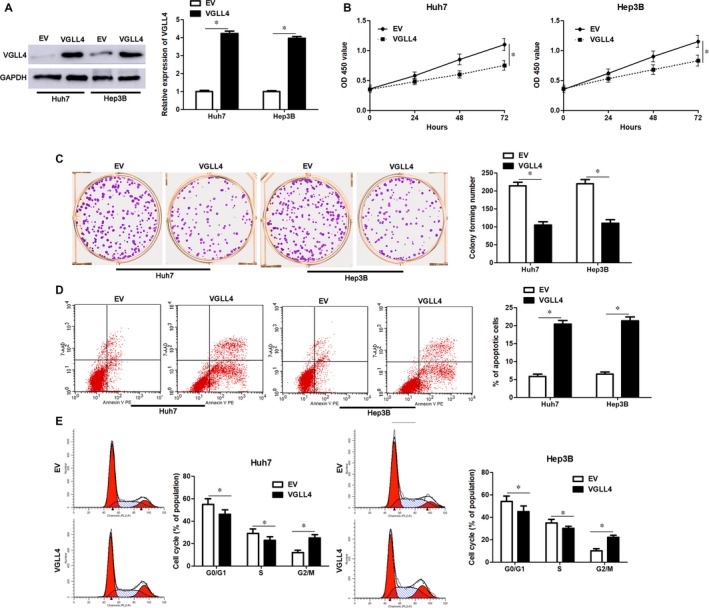
Vestigial like family member 4 (VGLL4) overexpression represses hepatocellular carcinoma (HCC) cell growth. A, pcDNA3.1‐VGLL4 or empty vector (EV) was transfected into Huh7 and Hep3B cells and immunoblotting was performed to detect the expression of VGLL4 protein. B, CCK‐8, (C) colony formation, (D) flow cytometry‐based apoptosis detection and (E) cell cycle distribution analysis was performed to determine the proliferation, apoptosis and cell cycle progression of HCC cells. n = three independent repeats, *P < 0.05 by *t* test or ANOVA

**Figure 6:**
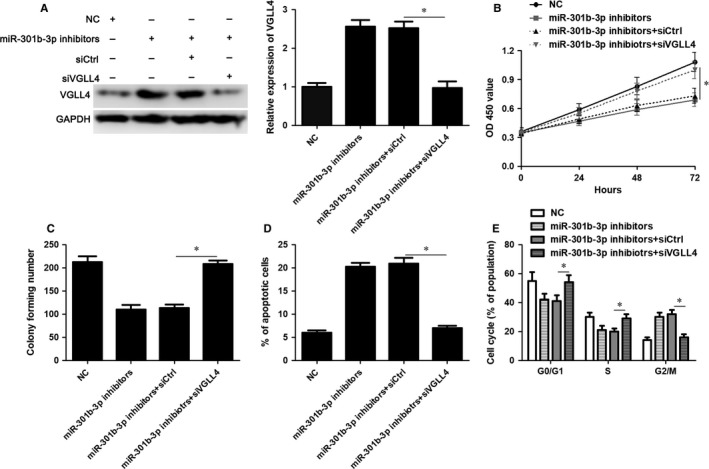
Vestigial like family member 4 (VGLL4) silencing rescues miR‐301b‐3p knockdown‐induced inhibition of proliferation, G2/M phase arrest and apoptosis of Huh7 cells. A, VGLL4 was knocked down by a specific siRNA in Huh7 cells with miR‐301b‐3p knockdown and immunoblotting was performed to detect VGLL4 protein. B, CCK‐8, (C) colony formation, (D) flow cytometry‐based apoptosis detection and (E) cell cycle distribution analysis was performed to determine the proliferation, apoptosis and cell cycle progression of Huh7 cells transfected with corresponding vectors. n = three independent repeats, *P < 0.05 by ANOVA

VGLL4 expression was restored by transfecting pcDNA3.1‐VGLL4 into Huh7 and Hep3B cells (*P* < 0.05, Figure 5A). VGLL4 restoration markedly inhibited cell proliferation, led to G2/M phase arrest and promoted apoptosis of Huh7 and Hep3B cells (*P* < 0.05, Figure 5B‐E), which was consistent with the effects of miR‐301b‐3p knockdown. To further study whether VGLL4 participates in miR‐301b‐3p knockdown‐induced inhibition of proliferation, G2/M phase arrest and apoptosis of HCC cells, VGLL4 was knocked down by a specific siRNA in Huh7 cells with miR‐301b‐3p knockdown (*P* < 0.05, Figure 6A). Silencing of VGLL4 promoted the proliferation and cell cycle progression of Huh7 cells with miR‐301b‐3p knockdown (*P* < 0.05, Figure 6B‐D). Moreover, knockdown of VGLL4 reversed miR‐301b‐3p silencing‐induced apoptosis of Huh7 cells (*P* < 0.05, Figure 6E). Thus, miR‐301b‐3p facilitated HCC cell proliferation, cell cycle progression and apoptosis resistance by targeting VGLL4.

## 4 DISCUSSION

The aberrant expression of miRNAs has been found to be a frequent event in human cancer. Overexpression of miR‐301b‐3p has been confirmed in various types of cancer.^14^, ^16^, ^17^, ^19^, ^20^, ^21^, ^23^ However, the expression pattern of miR‐301b‐3p in HCC was still unclear. This study found that miR‐301b‐3p was over‐expressed in HCC. Previous studies have revealed that several mechanisms are implicated in the aberrant expression of miR‐301b‐3p. For instance, hypoxia is a strong inducer for the overexpression of miR‐301b‐3p in lung cancer^21^, ^28^ and prostate cancer.^29^, ^30^ However, a recent study reports that aberrant promoter methylation leads to down‐regulation of miR‐130b~301b cluster in prostate cancer.^22^ Moreover, long non‐coding RNA lung cancer associated transcript 1 inversely regulates miR‐301b by acting as a competing endogenous RNA in hepatoblastoma.^31^ Thus, it is worth to further study the mechanism that contributes to the overexpression of miR‐301b‐3p in HCC. Increased expression of miR‐301b‐3p indicates poor survival of patients with pancreatic cancer.^16^ Moreover, the expression of miR‐301b‐3p in high‐grade ovarian serous carcinoma (HGS‐OvCa) with metastasis is significantly higher than that in HGS‐OvCa without metastasis, and its overexpression predicts poor clinical outcomes.^32^ Here, we found that high miR‐301b‐3p level was positively correlated with large tumour size and advanced TNM stages. Moreover, both our data and TCGA data demonstrated that HCC patients with high miR‐301b‐3p level had a significant poorer overall survival compared to patients in low miR‐301b‐3p group. Thus, miR‐301‐3p has potential as a prognostic marker for HCC.

Our previous studies have suggested that miRNAs are critical drivers or suppressors for the development and progression of HCC.^6^, ^10^, ^12^, ^25^, ^33^ For example, miR‐194‐5p functions as a tumour suppressor through inhibiting the tumour growth of HCC by targeting forkhead box A1.^6^ Down‐regulation of miR‐1296 contributes to migration, invasion and EMT of HCC cells by activating SRSF protein kinase 1‐mediated PI3K/AKT pathway.^12^ In this study, miR‐301b‐3p knockdown inhibited cell proliferation, led to G2/M arrest and induced apoptosis of HCC cells. In vivo experiments also demonstrated that miR‐301b‐3p silencing repressed tumour growth of HCC in mice. Recent studies have found the oncogenic role of miR‐301b‐3p in other tumour models. miR‐301b‐3p enhances migration and invasion of HGS‐OvCa, bladder cancer, hepatoblastoma and pancreatic cancer cells.^15^, ^20^, ^31^, ^32^ Furthermore, miR‐301b‐3p promotes the proliferation and inhibits apoptosis of breast cancer, melanoma, hepatoblastoma, bladder cancer and lung cancer cells.^18^, ^21^, ^23^, ^31^, ^34^ All these studies support the oncogenic role of miR‐301b‐3p in human cancer. Previous studies have disclosed several targets of miR‐301b‐3p, such as cytoplasmic polyadenylation element binding protein 3 in HGS‐OvCa,^32^ CYLD in breast cancer,^18^ leucine rich repeats and immunoglobulin like domains 1 in melanoma,^23^ early growth response gene 1 in bladder cancer,^34^ Bim in lung cancer,^21^ NR3C2 and TP63 in pancreatic cancer,^15^, ^16^ and NDRG2 in prostate cancer.^29^ Here, we identified VGLL4 as a novel target of miR‐301b‐3p in HCC. VGLL4 expression is reduced and has been verified as a tumour suppressor in HCC.^35^ Adenovirus‐mediated VGLL4 suppresses cell proliferation, causes G2/M phase arrest and enhances apoptosis of HCC cells, and restrains tumour growth of HCC in vivo.^36^ In this study, miR‐301b‐3p knockdown increased the abundance of VGLL4 in HCC cells and luciferase reporter assay indicated that miR‐301b‐3p directed bond to 3′UTR of VGLL4. Moreover, the expression of VGLL4 mRNA was down‐regulated and inversely correlated with miR‐301b‐3p level in HCC tissues. Importantly, VGLL4 overexpression resulted in inhibition of proliferation, G2/M arrest and apoptosis of HCC cells. VGLL4 knockdown rescued miR‐301b‐3p knockdown‐induced growth arrest and apoptosis of HCC cells. Thus, our findings provide evidence that miR‐301b‐3p plays an oncogenic role in HCC, at least partly, via directly repressing VGLL4.

In summary, we found that miR‐301b‐3p was highly expressed in HCC and overexpression of miR‐301b‐3p indicated poor clinical outcome of patients. VGLL4 was a novel direct target of miR‐301b‐3p in HCC. Moreover, miR‐301b‐3p facilitated cell proliferation and cell cycle progression, and prevented apoptosis of HCC cells by repressing VGLL4.

## 5 CONCLUSIONS

To conclude, our study demonstrates that miR‐301b‐3p overexpression is a frequent event in HCC and indicates poor clinical outcome of patients. Functionally, miR‐301b‐3p knockdown represses HCC cell proliferation, results in G2/M phase arrest and induces apoptosis in vitro, and restrains tumour growth of HCC in vivo. VGLL4 is recognized as a novel direct target of miR‐301b‐3p and participates in miR‐301b‐3p‐induced tumour progression of HCC. These observations support the notion that miR‐301b‐3p may be a novel potential target for HCC therapy.

## CONFLICT OF INTEREST

All authors declare no conflict of interest.

## AUTHOR CONTRIBUTION

QX, LY and DH conceived and designed the experiments; YG, BY, QZ, ZX, LH and XL performed the experiments; YG, BY and QZ analysed the data; LL and JW contributed reagents/materials/analysis tools; GY and QX wrote the paper. All authors read and approved the final manuscript.

## Data Availability

The data that support the findings of this study are available from the corresponding author upon reasonable request.
