# Supplementary figures and images for: MicroRNA‐301b‐3p contributes to tumour growth of human hepatocellular carcinoma by repressing vestigial like family member 4
Source: J Cell Mol Med. 2019 Jun 17;23(8):5037–47. doi: 10.1111/jcmm.14361 (PMC6653225; doi:10.1111/jcmm.14361)

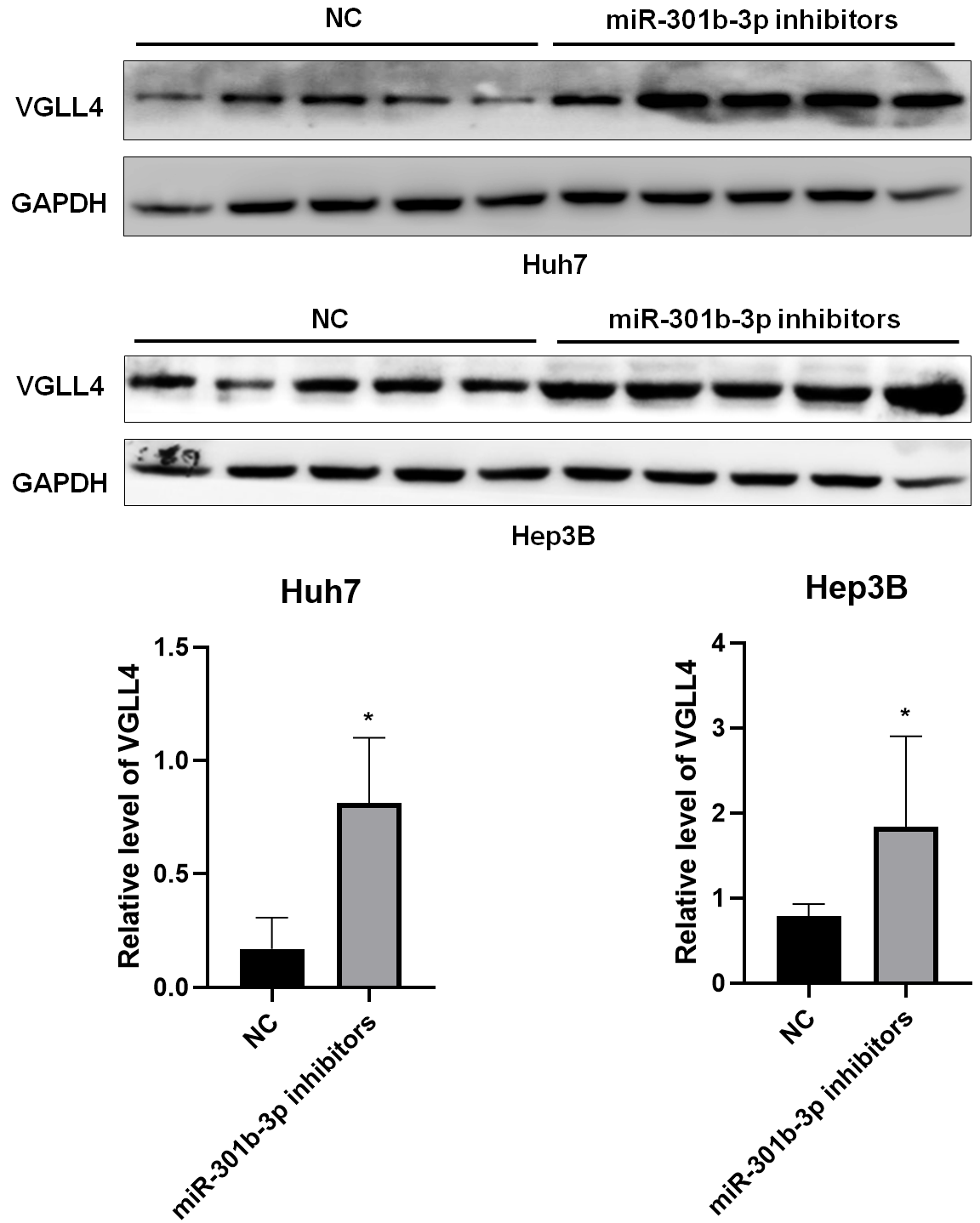

Supplement: Supplementary file 1 [file JCMM-23-5037-s001.tif]
